# Intrauterine vertical SARS‐CoV‐2 infection: a case confirming transplacental transmission followed by divergence of the viral genome

**DOI:** 10.1111/1471-0528.16682

**Published:** 2021-03-22

**Authors:** M Zaigham, A Holmberg, ML Karlberg, OK Lindsjö, L Jokubkiene, J Sandblom, AS Strand, O Andersson, SR Hansson, DG Nord, P Tannenberg

**Affiliations:** ^1^ Obstetrics & Gynaecology Department of Clinical Sciences Malmö Skåne University Hospital Lund University Lund Sweden; ^2^ Division of Infection Medicine Department of Clinical Sciences Lund Skåne University Hospital Lund University Lund Sweden; ^3^ Department of Infection Control Region Skåne Lund Sweden; ^4^ Unit for Laboratory Development and Technology Transfer Public Health Agency of Sweden Stockholm Sweden; ^5^ Neonatology Department of Clinical Sciences Lund Lund University Skåne University Hospital Malmö Sweden; ^6^ Department of Clinical Microbiology Lund University and Regional Laboratories Lund Sweden; ^7^ Obstetrics & Gynaecology Department of Clinical Sciences Lund Skåne University Hospital Lund University Lund Sweden; ^8^ Clinical Genetics and Pathology Laboratory Medicine Skåne University Hospital Lund Sweden; ^9^ Division of Clinical Genetics Department of Laboratory Medicine Lund University Lund Sweden; ^10^ Department of Paediatrics Skåne University Hospital Malmö Sweden; ^11^ Paediatric Cardiology Department of Clinical Sciences Lund Skåne University Hospital Lund University Lund Sweden

## Abstract

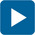
This article includes Author Insights, a video abstract available at https://vimeo.com/bjog/authorinsights16682

## Case

A 27‐year‐old woman (gravida 2, para 1) was transported to the regional university hospital in gestational week (GW) 34^+4^ due to a 3‐day history of fever, abdominal pain and reduced fetal movements. She had developed a dry cough 1 day prior to the admission (Figure S1).

The woman, was slightly overweight (body mass index 27 kg/m^2^) but otherwise healthy. She had normal antenatal check‐ups and an obstetric ultrasound at GW 32^+2^ showed a normal fetal weight deviation of +8%.^1^

At admission, the patient was promptly isolated in a negative pressure room at the delivery unit and standard operating procedures and personal protective equipment (PPE) were used.^2^ A combined nasopharynx (NPH) throat swab for SARS‐CoV‐2 using real time reverse transcriptase quantitative polymerase chain reaction (RT qPCR) was obtained and normal vital parameters (apart from 38.3ºC fever) were registered. The admission cardiotocograph (CTG) test showed reduced baseline variability, absence of accelerations with recurrent prolonged, and late decelerations (Figure S2). In light of the pathological CTG pattern, the obstetric team made the prompt decision to deliver the patient by an immediate caesarean section (CS). An uncomplicated CS was performed in an operating theatre with negative pressure, in line with the international recommendations for COVID‐19.^2^ The total blood loss was 200 ml. There was a normal amount of amniotic fluid and were no signs of meconium staining or premature rupture of the amniotic membranes.

The neonate showed no initial signs of spontaneous breathing and was ventilated by neonatal staff in a separate room. A maximum of 80% supplemental oxygen was needed to maintain adequate saturation. At 6 minutes of age, the neonate established spontaneous breathing and continuous positive airway pressure (5 cm H_2_O) was maintained for an additional 24 minutes, whereafter further ventilatory support was not needed. At 1 minute of age, the neonate had an Apgar score of 1 (heart rate = 1, remaining items = 0), at 5 minutes of age Apgar 4 (heart rate = 2, muscular tonus = 1, reflex irritability = 1, remaining items = 0), and at 10 minutes of age Apgar 8 (heart rate = 2, respiratory activity = 2, skin colour = 1, muscular tonus = 2, reflex irritability = 1). Validated umbilical cord blood gases^3^ showed a cord arterial pH of 7.20 and a venous pH of 7.22. Cord arterial lactate was 11 mmol/l and cord venous lactate 10.1 mmol/l. Figure S1 illustrates the timeline of events for mother and child.

After the CS, the mother was isolated in the postpartum ward; the NPH/throat swab taken upon admission returned positive for SARS‐CoV‐2. Analysis of maternal blood was also RT qPCR‐positive for SARS‐CoV‐2. Serology from the day of delivery revealed that the mother was weakly positive for immunoglobulin (Ig) M and negative for IgG. Along with lymphocytopenia (0.7 × 10^9^/l) and thrombocytopenia (98 x 10^9^/l); inflammatory markers including c‐reactive protein (36 mg/l), ferritin (340 mcmol/l) and lactate dehydrogenase (9.5 mckat/l) were found to be elevated. The clinical condition of the mother improved and she was discharged 4 days after delivery. Thromboprophylaxis (Tinzaparin 4500 IE subcutaneously once daily) was prescribed for 6 weeks postpartum in accordance with national guidelines in place at the time.^4^ By day 11 postpartum, the mother was seropositive for anti‐SARS‐CoV‐2 IgM and IgG. Breast milk analysed day 14 postpartum was RT qPCR‐negative for SARS‐CoV‐2 and, further, at day 35 postpartum, the breast milk was negative for anti‐SARS‐CoV‐2 total immunoglobulin.

The neonate in this case had no contact with any family member, including the mother, for the first 60 hours of life. As neither skin‐to‐skin care nor any other contact with the mother occurred, the neonate was regarded as non‐infected. In accordance with national guidelines at the time,^4^ the neonate was tested for COVID‐19 using a NPH swab 48 hours after delivery. This test returned positive for SARS‐CoV‐2 and the neonate was then regarded as contagious. Infection control routines were initiated to investigate a potential COVID‐19 breakout at the neonatal ward and to rule out the possibility of postpartum transmission. All staff that had tended to the neonate (*n* = 27) and all nearby patients (*n* = 4) were tested by NPH swab; SARS‐CoV‐2 RT qPCR returned negative in all cases (data not shown). Symptom surveillance in this group was continued for a further 14 days but no COVID‐19‐positive cases were discovered during this time.

The neonate was transferred and united with the mother in the postpartum ward isolation room at day of life (DOL) 3 (60 hours after birth). Breastfeeding was thereafter initiated and the neonate did not receive any breastmilk before this time point. Repeated RT qPCR analyses showed the lowest neonatal CT‐value at DOL 5, whereafter a gradual increase was seen. By DOL 20, SARS‐CoV‐2 was not detectable in NPH or throat swabs (Table S2). Serology revealed that the neonate was anti‐SARS‐CoV‐2 IgG‐negative at DOL 7 (IgM not analysed due to lack of material). At DOL 14, IgM was positive and IgG still negative, and at DOL 20, the neonate was both IgM‐ and IgG‐seropositive.

### Viral genome sequencing

To determine the genetic clade and to investigate fully the viral genetic similarities, virus isolates from the mother (NPH/throat swab obtained on the day of delivery) and neonate (NPH swab obtained at 48 hours of age, labelled DOL 2, and further at DOL 5) as well as from placental tissue, were sent to the Public Health Agency of Sweden for whole‐genome sequencing. Next‐generation sequencing of samples produced several full‐length 29 903 bp, SARS‐CoV‐2 genomes, all belonging to the genetic clade 20B/GR/B.1.1 (Table S3).^5^ All four sequences showed high identity. Further sequencing data analysis identified 12 variant positions in the sequences from isolates of the mother and placenta compared with the SARS‐CoV‐2 reference genome (NC_045512). These variants were also present in the sequences of the neonatal isolates. Notably, an additional variant, A107G, was identified in the neonate samples but was only present in 67 and 80%, respectively, of the sequences.

### Placental pathology

**Figure 1:**
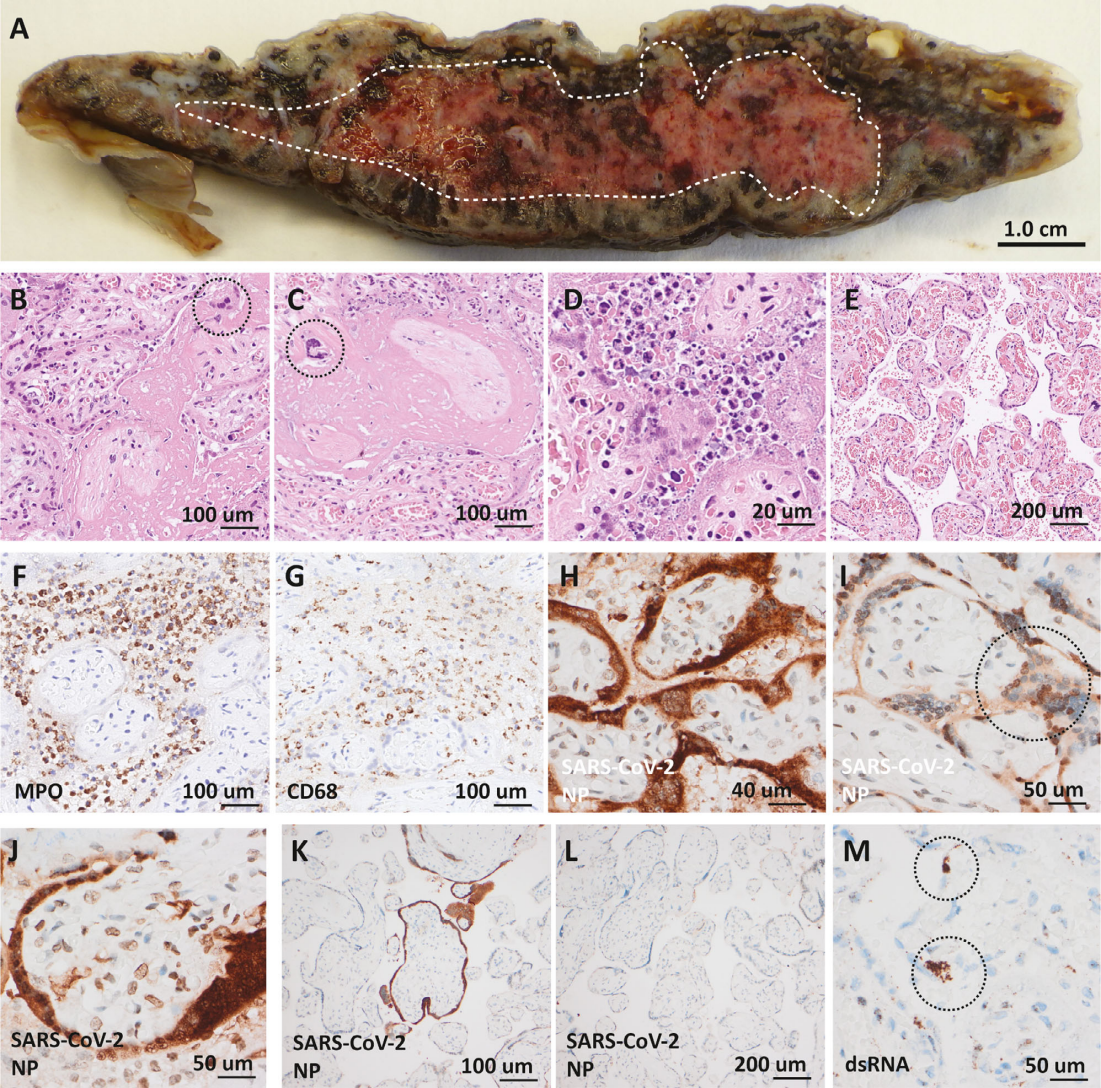
Placental pathology. (A) Transected placenta with confluent accumulation of fibrinoid demarcated (white broken line). (B,C) Massive intervillous fibrinoid deposition surrounding denuded villi with extravillous syncytiotrophoblasts (circles) located in the fibrinoid. (D) Acute intervillositis with karyorrhectic neutrophils in the intervillous space and degeneration of the villous trophoblast layer. (E) Representative region of chorangiosis. (F,G) Immunohistochemical staining for myeloperoxidase (MPO) and CD68 with positivity in inflammatory cells in areas of intervillositis. (H‐J) Severe acute respiratory syndrome coronavirus‐2 (SARSCoV‐2) nucleoprotein (NP) detected in nucleus and (circle in I) and cytoplasm in villous trophoblasts and syncytiotrophoblasts as well as in the nucleus of villous stromal cells (J) in areas of intervillositis. (K,L) Areas without intervillositis showed absent or focal staining for SARS‐CoV‐2 nucleoprotein of villi. (M) Double‐stranded RNA (dsRNA) detected in villous trophoblasts and syncytiotrophoblasts (circles).

The placenta was easily detached from the uterus during the CS. The remaining umbilical cord stump had a central insertion, was 9 cm long with a diameter of 1 × 1.5 cm and contained three vessels. The membranes had normal colour without signs of meconium staining. The trimmed weight of the placental disc was 342 g, within the 10th–90th percentile for GW 34^+0^ to 34^+6^.^6^ At gross sectioning, fibrinoid depositions were evident as glistening white‐grey‐pink confluent lesions, encompassing approximately 50% of the total placental volume (Figure 1A).

Microscopic examination confirmed the presence of confluent intervillous fibrinoid depositions accompanied by denudation of the villi from trophoblasts and syncytiotrophoblasts with dislocated syncytiotrophoblasts visible in the fibrinoid (Figure 1B,C). There were multiple regions of dense intervillous infiltrates of neutrophilic granulocytes and macrophages (Figure 1D). The areas devoid of intervillous fibrinoid depositions frequently showed chorangiosis (Figure 1E). Immunohistochemistry confirmed that the inflammatory cell component of the intervillisitis was dominated by myeloperoxidase‐positive granulocytes and CD68‐positive macrophages with sparse amounts of CD3‐ and CD20‐positive lymphocytes (Figure 1F,G).

Immunohistochemical detection of SARS‐CoV‐2 nucleoprotein was strongly positive in the cytoplasm and nucleus of villous cytotrophoblasts and syncytiotrophoblasts in areas with intervillositis and fibrinoid depositions, with some positive staining in the villous stromal cells (Figure 1H–J). In contrast, SARS‐CoV‐2 nucleoprotein staining was focal or absent in most but not all areas devoid of intervillositis (Figure 1K,L). Additionally, presence of ribonucleic acid (RNA) virus was confirmed in both cytotrophoblasts and syncytiotrophoblasts by *in situ* staining for double‐stranded RNA (Figure 1M). There were no signs of villitis or inflammation in the membranes or umbilical cord. Immunohistochemistry for SARS‐CoV‐2 nucleoprotein was absent or showed faint signal in the amniotic membranes and the fetal chorionic vessels.

## Discussion

Vertical transmission is one of the major complications of viral diseases during pregnancy.^7^ Pregnant women are more likely to need intensive care treatment related to COVID‐19 as compared with non‐pregnant women of reproductive age.^8^ COVID‐19 infection has also been associated with a higher rate of preterm birth, pre‐eclampsia, CS, fetal vascular malperfusion, premature fetal membrane rupture and perinatal death.^9^, ^10^ A number of reports have suggested vertical transmission^11^ but, to the best of our knowledge, only Vivanti et al.,^12^ Fenizia et al.^13^ and Correia et al.^14^ have convincingly reported cases of transplacental SARS‐CoV‐2 transmission (Table S1).

Several studies have found SARS‐CoV‐2 in placental tissue, amniotic fluid and cord blood;^15^, ^16^, ^17^ however, vertical transmission seems to be a rare complication of COVID‐19 in pregnancy. SARS‐CoV‐2 may be physically blocked by the placental barrier defence mechanisms, either combatted by immune‐regulatory molecular pathways or, in the case of placental infection, immunomodulatory mechanisms may soften the cytokine storm associated with severe COVID‐19 disease. This reduction in cell and tissue damage has been postulated potentially to reduce the risk of SARS‐CoV‐2 transmission to the fetus.^18^ The placenta is therefore of key interest in understanding perinatal transmission. For SARS‐CoV‐2, angiotensin‐converting enzyme 2 (ACE2) is the undisputed receptor for cellular entry. Co‐expression of the protease, TMPRSS2, is also required to cleave the spike (S) protein on SARS‐CoV‐2, mediating ACE2 binding. Studies have shown low levels of ACE2 and TMPRSS2 mRNA in the syncytiotrophoblast and extravillous trophoblast cells of the placenta^15^, ^19^ and, due to negligible co‐transcription of ACE2 and TMPRSS2 in the placenta, it has been argued that ACE2 is not a likely path of vertical transmission for SARS‐CoV‐2.^20^ In contrast, receptors for Zika virus and cytomegalovirus, which cause congenital infections, are abundant in placental cell types. To the best of our knowledge, no studies have demonstrated SARS‐CoV‐2 cellular entry via ACE2 binding or replication within placental cells.^21^

Other studies have suggested that placental inflammation may play a central role in the transmission of SARS‐CoV‐2. Lye et al.^22^ reported that ACE2 expression was increased in placentas that experienced chorioamnionitis. The authors proposed the possibility of vertical transmission by way of SARS‐CoV‐2‐carrying immune cells infiltrating placentas complicated with chorioamnionitis. Antibodies can also facilitate the transport of viruses across the placenta by Fc receptor binding. SARS‐CoV‐2 infection of placental macrophages (Hofbauer cells) has only been identified in a single patient from one clinical report, suggesting that this may be a rare mechanism of SARS‐CoV‐2 transmission.^23^ There is additional evidence, as described by Yoel Sadovsky et al.,^24^ on how miRNA in the placenta can protect against viral infections.

In the current study, viraemia may have led to more severe maternal disease with rapid deterioration of placental function secondary to inflammation. Viraemia in the blood is rare. According to Wang et al.,^25^ SARS‐CoV‐2 RNA was found in only 1% of blood samples taken from COVID‐19 patients. It may therefore be argued that the placenta is highly active in preventing SARS‐CoV‐2 vertical transmission and although the exact mechanisms of placental defense against the virus are yet to be elucidated, inflammation may play a central role.

The mother presented with classic COVID‐19 symptoms including fever and a dry cough,^26^ but abdominal pain and reduced fetal movements were also reported. Similar to previous reports, we observed that the clinical condition of the mother improved rapidly after delivery.^27^, ^28^ The mother also presented with elevated concentrations of several acute phase proteins including ferritin, procalcitonin and c‐reactive protein, indicating systemic inflammation.^29^ In addition, at the time of delivery, SARS‐CoV‐2 RNA was found in the maternal blood and RT qPCR indicated the highest viral load within the placenta. RT qPCR does not produce an exact quantification of viral load, as different materials are analysed. However, the cycle threshold (CT) values were clearly the lowest in the placental specimen and histopathological placental analyses indicated high levels of SARS‐CoV‐2. Viral protein was found in the villous cytotrophoblasts and in the syncytiotrophoblasts, and massive perivillous fibrin deposits covered over 50% of the placenta. The placental histopathological changes seen in this case are similar to several previous reports on SARS‐CoV‐2, as well as SARS‐CoV‐1 and MERS‐CoV.^12^, ^23^, ^30^, ^31^

The neonate in the current case suffered from transient asphyxia attributed to intrauterine hypoxia secondary to placental dysfunction. This was signalled by the pathological CTG result, and validated umbilical cord blood gases revealed a cord arterial and venous pH below normal median reference values for 34 weeks of gestational age.^3^ More notably, the neonate had abnormally high cord arterial and venous lactate values, which clearly indicated a hypoxic insult.^32^ Following initial resuscitation, only standard supportive care of prematurity was needed. No evident signs of COVID‐19 were observed and repeated RT qPCR testing revealed the lowest CT‐values at DOL 5, suggestive of the highest viral load in the upper respiratory tract at this time point, as a lower CT level implies a greater amount of SARS‐CoV‐2 nucleic acid. The CT‐values later increased and by DOL 20, SARS‐CoV‐2 RNA was not detectable. Consistent with the observed viral clearance, neonatal IgM and IgG seroconversion was found. Previous knowledge of immunoglobulin transfer during pregnancy along with new data from the current COVID‐19 pandemic confirm that anti‐SARS‐CoV‐2 IgG can pass through the placental barrier, but IgM does not.^33^, ^34^ In the current case, maternal serum was weakly positive for IgM and negative for IgG on the day of delivery. Further, the neonate was seronegative for IgG at DOL 7 (IgM not tested due to lack of material) and we conclude that transplacental transfer of anti‐SARS‐CoV‐2 immunoglobulin was not likely and that the neonate seroconverted by its own means. The possibility of the neonate acquiring COVID‐19 postpartum was ruled out by vigorous testing of all staff that had been in contact with the neonate during the first 48 hours of life, as well as surrounding patients and their attendees. Secondary symptom surveillance for 2 weeks revealed no new cases.

To determine fully, viral genome similarities between the mother, neonate and the placenta, whole‐genome sequencing was performed. All four isolates revealed 29 903 bp SARS‐CoV‐2 genomes, belonging to the genetic clade 20B/GR/B.1.1. Further analysis of the sequencing data showed that the isolate from mother and placenta had 11 single‐nucleotide polymorphisms (SNPs) and one multiple‐nucleotide polymorphism (MNP) difference compared with the reference Wuhan genome of SARS‐CoV‐2 (Figure S3). Interestingly, the two neonatal isolates, from DOL 2 and DOL 5, both had a mixed population of the virus. In addition to a population of the virus with the same genotype as the isolates from the mother and placenta, the neonate isolates contained another population of virus (80% identical to maternal isolates on DOL 2 and 67% in DOL 5) with an additional SNP, e.g. A107G. Intrapatient genetic variation has previously been described in both MERS‐CoV and SARS‐CoV‐2.^35^, ^36^ To the best of our knowledge, this is the first case of ongoing genetic change in neonatal COVID‐19 in the unique setting of intrauterine transmission.

The SARS‐CoV‐2 genome has been reported to have an evolutionary rate of around 8 × 10^−4^ substitutions per site per year.^37^, ^38^ Although rapid compared with its host’s evolutionary rate or bacteria, this is considered slow for an RNA virus. On the basis of this and previous studies in literature, we did not expect the genome to diverge. As such, even the small divergencevide seen between the two patients is worth noting. There are several explanations for the genetic divergence of the virus in the current case. One is genetic drift, i.e. a genetic variant that happened to spread without being actively selected for. It may have occurred by random mutation in the placenta with replication in parallel with the original variant. We have no data distinguishing drift from selection. This would require functional studies and more cases where this variant is demonstrated to be enriched in the placenta compared with the mother. Secondly, and more likely, transfer from mother to neonate may have spurred evolution, due to change in the environment. Overall, however, all virus isolates from mother, neonate and the placenta displayed a clear similarity and shared a majority of the SNPs.

Given these genetic findings and the series of events presented above, along with the marked placental pathology and the high viral load, it can be concluded that the neonate was infected *in utero*. Two main clinical lessons can be learnt from the current case:

- intrauterine vertical transmission is an uncommon complication of COVID‐19 during pregnancy which may lead to placental dysfunction and clinical consequences for the newborn
- intrauterine SARS‐CoV‐2 transmission may not necessarily lead to severe neonatal outcome.

### Disclosure of interests

None declared. Completed disclosure of interests forms are available to view online as supporting information.

### Contribution to authorship

MZ, AMH and PT conceived the project, performed the literature search, prepared the tables, figures, merged and interpreted all the data and wrote the manuscript draft. LJ, MZ and AMH managed the mother. MZ interpreted the maternal clinical picture and laboratory tests. PT, JS and OA managed the neonate, and interpreted the neonatal clinical picture and laboratory tests. ASS interpreted the SARS‐CoV‐2 diagnostic data. MLK and OKL performed the whole‐genome sequencing and data analysis. SRH, DGN, MLK and OKL helped in data interpretation and revision of the manuscript. DGN performed the pathological examination, prepared the figures and co‐authored the text. All authors critically reviewed the manuscript for important intellectual content and approved it in its final version.

### Details of ethics approval

The mother and father have provided written informed consent for publication, available upon request. The case study was performed in agreement with principles of the Declaration of Helsinki.

### Funding

MZ was supported by research grants from Region Skåne and the Medical Faculty, Lund University, Sweden (ALF). The funders had no role in study design, data collection and analysis, decision to publish, or preparation of the manuscript.

### Acknowledgements

The authors would like to thank the COVID‐19 in Pregnancy and Early Childhood (COPE) study for allowing the use of a maternal blood sample, Dr Yang De Marinis for donating a COVID‐19 serological kit from ZetaGene, Dr Ylva Lindroth and Dr David Nygren from Department of Clinical Microbiology, Skåne University Hospital Lund, Sweden. The authors would like to acknowledge Mia Brytting at the Public Health Agency of Sweden for assistance in the interpretation of sequencing results as well as Niklas Edner for preliminary clinical consulting and facilitation of whole‐genome sequencing. The authors also gratefully acknowledge the researchers from the originating laboratories responsible for obtaining the specimens and the submitting laboratories where genetic sequence data were generated and shared via the GISAID initiative, on which this research is based.

## Supporting information


**Figure S1.** Timeline of events for mother and neonate.

**Figure S2.** Admission cardiotocograph (CTG) of the fetal heart rate.

**Figure S3.** Phylogenetic tree of closely related sequences; the lowest two branches in the tree represent the mother (20‐08953), the placenta (20‐52814) and the neonate on DOL 2(20‐08955) and DOL 5 (20‐08954).

**Table S1.** Previous confirmed cases of intrauterine SARS‐CoV‐2 transmission.

**Table S2.** Real time reverse transcriptase quantitative polymerase chain reaction (PCR) analyses with cycle threshold (CT) values for severe acute respiratory syndrome coronavirus‐2 (SARS‐CoV‐2) in maternal, placental and neonatal samples.

**Table S3.** Variant analysis and annotation of whole‐genome sequencing data from virus isolates obtained from the mother and placenta at delivery and from the neonate at day of life (DOL) 2 and DOL 5.

Supplementary Material

Supplementary Material

Supplementary Material

Supplementary Material

Supplementary Material

Supplementary Material

Supplementary Material

Supplementary Material

Supplementary Material

Supplementary Material

Supplementary Material

Supplementary Material

## Data Availability

The datasets generated during and/or analysed during the current study are available from the corresponding author on reasonable request.

## References

[1] Marsál K , Persson PH , Larsen T , Lilja H , Selbing A , Sultan B . Intrauterine growth curves based on ultrasonically estimated foetal weights. Acta Paediatr 2020;85:843–8. 10.1111/j.1651-2227.1996.tb14164.x 8819552

[2] Coronavirus (COVID‐19) Infection in Pregnancy . Information for healthcare professionals (online) Version 7: Published Thursday 9 April 2020. [https://www.rcog.org.uk/globalassets/documents/guidelines/2020‐04‐09‐coronavirus‐covid‐19‐infection‐in‐pregnancy.pdf]. Accessed 25 September 2020.

[3] Zaigham M , Källen K , Olofsson P . Gestational age‐related reference values for Apgar score and umbilical cord arterial and venous pH in preterm and term newborns. Acta Obstet Gynecol Scand 2019;98:1618–23. 31318453 10.1111/aogs.13689

[4] Swedish Society for Obstetrics and Gynecology and Swedish Neonatal Society (Internet) recommendations for care of pregnant women and neonates born to women with confirmed or suspected COVID‐19. (online)Version 2, updated 2020‐04‐05. [https://neo.barnlakarforeningen.se/wp‐content/uploads/sites/14/2020/03/Rekommendation‐om‐handläggning‐av‐gravida‐och‐barn‐till‐kvinnor‐med‐verifieradelsannolik‐Covid‐19_ver‐2_200405.pdf]. Accessed 9 October 2020.

[5] Global Initiative on Sharing All Influenza Data (GISAID) . Genomic epidemiology of hCoV‐19 EpiCoV (online) [https://www.epicov.org/]. Accessed 9 October 2020.

[6] Kraus F , Redline R , Gersell D , Nelson M , Placental DJ . Placental Pathology. Silver Spring: American Registry of Pathology; 2005.

[7] Pereira L . Congenital viral infection: traversing the uterine‐placental interface. Annu Rev Virol 2018;5:273–99. 30048217 10.1146/annurev-virology-092917-043236

[8] Ortiz‐Prado E , Simbana‐Rivera K , Gomez‐Barreno L , Rubio‐Neira M , Gauman L , Kyriakidis N , et al. Clinical, molecular, and epidemiological characterization of the SARS‐CoV‐2 virus and the Coronavirus Disease 2019 (COVID‐19), a comprehensive literature review. Diagn Microbiol Infect Dis 2020;98:115094. 32623267 10.1016/j.diagmicrobio.2020.115094 PMC7260568

[9] Allotey J , Stallings E , Bonet M , Yap M , Chatterjee S , Kew T , et al. Clinical manifestations, risk factors, and maternal and perinatal outcomes of coronavirus disease 2019 in pregnancy: living systematic review and meta‐analysis. BMJ 2020;370:3320. 10.1136/bmj.m3320 PMC7459193 32873575

[10] Dubey P , Reddy SY , Manuel S , Dwivedi AK . Maternal and neonatal characteristics and outcomes among COVID‐19 infected women: an updated systematic review and meta‐analysis. Eur J Obstet Gynecol Reprod Biol 2020;252:490–501. 32795828 10.1016/j.ejogrb.2020.07.034 PMC7373687

[11] Molloy EJ , Lavizzari A , Klingenberg C , Profit J , Zupancic J , Davis A , et al. Neonates in the COVID‐19 pandemic. Pediatr Res 2020. https://doi.org/10.1038/s41390‐020‐1096‐y [Epub ahead of print]. 10.1038/s41390-020-1096-y 32746446

[12] Vivanti AJ , Vauloup‐Fellous C , Prevot S , Zupan V , Suffee C , Cao J , et al. Transplacental transmission of SARS‐CoV‐2 infection. Nat Commun 2020;11:3572. 32665677 10.1038/s41467-020-17436-6 PMC7360599

[13] Fenizia C , Biasin M , Cetin I , et al. Analysis of SARS‐CoV‐2 vertical transmission during pregnancy. Nat Commun 2020;11:5128. 33046695 10.1038/s41467-020-18933-4 PMC7552412

[14] Correia CR , Marçal M , Vieira F , Santos E , Novais C , Maria AT , et al. Congenital SARS‐CoV‐2 infection in a neonate with severe acute respiratory syndrome. Pediatr Infect Dis J 2020;39:e439–e443. 33060519 10.1097/INF.0000000000002941

[15] Algarroba GN , Hanna N , Rekawek P , Vahanian S , Khullar P , Palaia T , et al. Confirmatory evidence of the visualization of severe acute respiratory syndrome coronavirus 2 invading the human placenta using electron microscopy. Am J Obstet Gynecol 2020;223:953–4. 10.1016/j.ajog.2020.08.106 PMC7453223 32866527

[16] Hosier H , Farhadian S , Morotti R , Deshmukh U , Lu‐Culligan A , Campbell K , et al. SARS‐CoV‐2 infection of the placenta. J Clin Invest 2020;130:4947–53. 32573498 10.1172/JCI139569 PMC7456249

[17] Patane L , Morotti D , Giunta M , Sigismondi C , Piccoli M , Frigerio L , et al. Vertical transmission of coronavirus disease 2019: severe acute respiratory syndrome coronavirus 2 RNA on the fetal side of the placenta in pregnancies with coronavirus disease 2019‐positive mothers and neonates at birth. Am J Obstet Gynecol 2020;2:100145. 10.1016/j.ajogmf.2020.100145 PMC7233206 32427221

[18] Kreis NN , Ritter A , Louwen F , Yuan J . A Message from the human placenta: structural and immunomodulatory defense against SARS‐CoV‐2. Cells 2020;9:1777. 10.3390/cells9081777 PMC7465902 32722449

[19] Ashary N , Bhide A , Chakraborty P , Colaco S , Mishra A , Chhabria K , et al. Single‐cell RNA‐seq identifies cell subsets in human placenta that highly expresses factors driving pathogenesis of SARS‐CoV‐2. Front Cell Dev Biol 2020;8:783. 32974340 10.3389/fcell.2020.00783 PMC7466449

[20] Pique‐Regi R , Romero R , Tarca AL , Luca F , Xu Y , Alazizi A , et al. Does the human placenta express the canonical cell entry mediators for SARS‐CoV‐2? Elife 9 2020;9:e58716. 10.7554/eLife.58716 PMC7367681 32662421

[21] Moore KM , Suthar MS . Comprehensive analysis of COVID‐19 during pregnancy. Biochem Biophys Res Commun 2020;20:32241–5. 10.1016/j.bbrc.2020.12.064 PMC7759124 33384142

[22] Lye P , Dunk C , Zhang J , Wei Y , Nakpu J , Hamada H , et al. SARS‐CoV‐2 cell entry gene ACE2 expression in immune cells that infiltrate the placenta in infection‐associated preterm birth. MedRxiv 2020;20201590. 10.3390/cells10071724 PMC8303980 34359894

[23] Facchetti F , Bugatti M , Drera E , Tripodo C , Sartori E , Cancila V , et al. SARS‐CoV‐2 vertical transmission with adverse effects on the newborn revealed through integrated immunohistochemical, electron microscopy and molecular analyses of placenta. EBioMedicine 2020;59:102951. 32818801 10.1016/j.ebiom.2020.102951 PMC7430280

[24] Delorme‐Axford E , Sadovsky Y , Coyne CB . The placenta as a barrier to viral infections. Ann Rev Virol 2014;1:133–46. 26958718 10.1146/annurev-virology-031413-085524

[25] Wang W , Xu Y , Gao R , Lu R , Han K , Wu G , et al. Detection of SARS‐CoV‐2 in different types of clinical specimens. JAMA 2020;323:1843–4. 32159775 10.1001/jama.2020.3786 PMC7066521

[26] Zaigham M , Andersson O . Maternal and perinatal outcomes with COVID‐19: a systematic review of 108 pregnancies. Acta Obstet Gynecol Scand 2020;99:823–9. 32259279 10.1111/aogs.13867 PMC7262097

[27] Ronnje L , Länsberg JK , Vikhareva O , Hansson SR , Herbst A , Zaigham M . Complicated COVID‐19 in pregnancy: a case report with severe liver and coagulation dysfunction promptly improved by delivery. BMC Pregnancy Childbirth 2020;20:511. 32887569 10.1186/s12884-020-03172-8 PMC7472409

[28] Kolkova Z , Bjurström M , Länsberg JK , Svedas E , Hamer M , Hansson S , et al. Obstetric and intensive‐care strategies in a high‐risk pregnancy with critical respiratory failure due to COVID‐19: a case report. Case Rep Womens Health 2020;27:e00240. 32714844 10.1016/j.crwh.2020.e00240 PMC7340590

[29] Henderson LA , Canna S , Schulert G , Volpi S , Lee P , Kernan K , et al. On the alert for cytokine storm: immunopathology in COVID‐19. Arthritis Rheumatol 2020;72:1059–63. 32293098 10.1002/art.41285 PMC7262347

[30] Schoenmakers S , Snijder P , Verdijk R , Kuiken T , Kamphuis S , Koopman L , et al. SARS‐CoV‐2 placental infection and inflammation leading to fetal distress and neonatal multi‐organ failure in an asymptomatic woman. medRxiv. 10.1101/20020.06.08.20110437. Accessed 9 October 2020. PMC7798999 33367801

[31] Ng WF , Wong SF , Lam A , Mak YF , Lee KC , Chow KM , et al. The placentas of patients with severe acute respiratory syndrome: a pathophysiological evaluation. Pathology 2006;38:210–8. 16753741 10.1080/00313020600696280 PMC7131423

[32] Wiberg N , Källen K , Herbst A , Åberg A , Olofsson P . Lactate concentration in umbilical cord blood is gestational age‐dependent: a population‐based study of 17 867 newborns. BJOG 2008;115:704–9. 18410653 10.1111/j.1471-0528.2008.01707.x

[33] Kohler PF , Farr RS . Elevation of cord over maternal IgG immunoglobulin: evidence for an active placental IgG transport. Nature 1966;210:1070–1. 5950290 10.1038/2101070a0

[34] Zeng H , Xu C , Fan J , Tang Y , Deng Q , Zhang W , et al. Antibodies in infants born to mothers with COVID‐19 pneumonia. JAMA 2020;323:1848–9. 32215589 10.1001/jama.2020.4861 PMC7099444

[35] Park D , Huh H , Kim Y , Son D , Jeon H , Im E , et al. Analysis of intrapatient heterogeneity uncovers the microevolution of Middle East respiratory syndrome coronavirus. Cold Spring Harb Mol Case Stud 2016;2:a001214. 27900364 10.1101/mcs.a001214 PMC5111008

[36] Jary A , Leducq V , Malet I , Marot S , Klement‐Frutos E , Teyssou E , et al. Evolution of viral quasispecies during SARS‐CoV‐2 infection. Clin Microbiol Infect 2020;26:1560.e1–.e4. 10.1016/j.cmi.2020.07.032 PMC7378485 32717416

[37] Rambaut A . Phylodynamic Analysis. Virological. Published 6 March 2020. [http://virological.org/t/phylodynamic‐analysis‐176‐genomes‐6‐mar‐2020/356]. Accessed 5 January 2020.

[38] Su YCF , Anderson DE , Young BE , Linster M , Zhu F , Jayakumar J , et al. Discovery and genomic characterization of a 382‐nucleotide deletion in ORF7b and ORF8 during the early evolution of SARS‐CoV‐2. mBio 2020;11:e01610‐20. 32694143 10.1128/mBio.01610-20 PMC7374062

