# Supplementary figures and images for: Intrauterine vertical SARS‐CoV‐2 infection: a case confirming transplacental transmission followed by divergence of the viral genome
Source: BJOG. 2021 Mar 22;128(8):1388–94. doi: 10.1111/1471-0528.16682 (PMC8013698; doi:10.1111/1471-0528.16682)

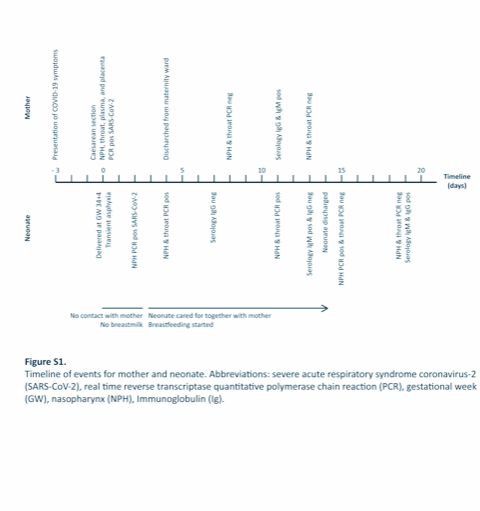

Supplement: Supplementary file 1 — Figure S1. Timeline of events for mother and neonate. [file BJO-128-1388-s008.tiff]

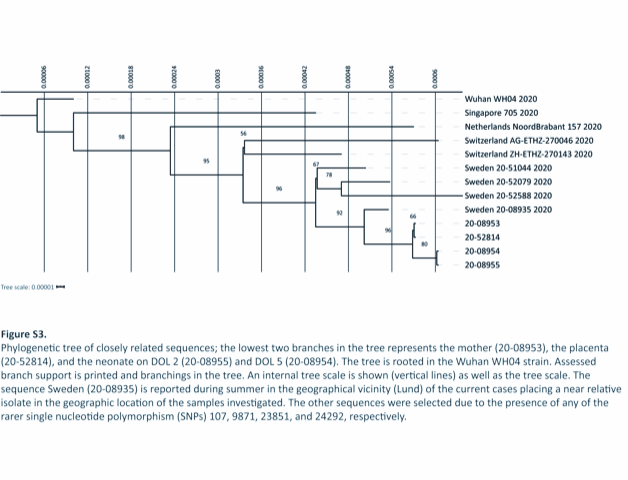

Supplement: Supplementary file 3 — Figure S3. Phylogenetic tree of closely related sequences; the lowest two branches in the tree represent the mother (20‐08953), the placenta (20‐52814) and the neonate on DOL 2(20‐08955) and DOL 5 (20‐08954). [file BJO-128-1388-s013.tiff]
