# Supplementary material for: Intrauterine vertical SARS‐CoV‐2 infection: a case confirming transplacental transmission followed by divergence of the viral genome
Source: BJOG. 2021 Mar 22;128(8):1388–94. doi: 10.1111/1471-0528.16682 (PMC8013698; doi:10.1111/1471-0528.16682)
Supplement: Supplementary file 2 — Figure S2. Admission cardiotocograph (CTG) of the fetal heart rate. [file BJO-128-1388-s006.docx]

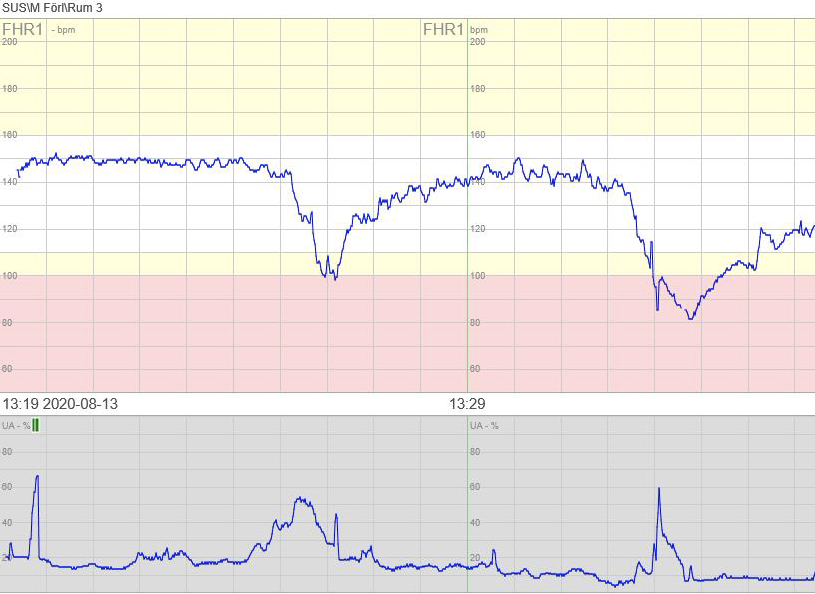


**Figure S2.**

Admission cardiotocograph (CTG) of the foetal heart rate. Normal foetal heart rate with reduced baseline variability, absence of accelerations and with recurrent prolonged and late decelerations. These findings are suggestive of a pathological category III foetal heart rate tracing which is strongly associated with adverse neonatal outcome. The CTG was recorded 8 minutes before the immediate-emergency caesarean section was performed. Paper velocity: 1 centimetre per minute.
