# Supplementary material for: Intrauterine vertical SARS‐CoV‐2 infection: a case confirming transplacental transmission followed by divergence of the viral genome
Source: BJOG. 2021 Mar 22;128(8):1388–94. doi: 10.1111/1471-0528.16682 (PMC8013698; doi:10.1111/1471-0528.16682)
Supplement: Supplementary file 4 — Table S1. Previous confirmed cases of intrauterine SARS‐CoV‐2 transmission. [file BJO-128-1388-s016.docx]

**Table S1.**

Previous confirmed cases of intrauterine SARS-CoV-2 transmission.

| Authors | Article type | Maternal NPH | Maternal  plasma | Placenta | Amniotic fluid | Cord blood | Neonatal plasma | Neonatal NPH | Gestational week at birth |
| --- | --- | --- | --- | --- | --- | --- | --- | --- | --- |
| Vivanti et al^12^ | Case report | PCR pos | PCR pos | PCR and IHC pos | PCR pos | n/a | PCR pos | PCR pos | 35 + 5 |
| Fenizia et al^13^ | Case series* | PCR pos | PCR pos | PCR pos | n/a | PCR pos | n/a | PCR pos | 34 + 4 |
| Correia et al^14^ | Case report | PCR pos | n/a | n/a | n/a | n/a | PCR pos | PCR pos | 34 + 2 |

Foot note:

* One confirmed case of intrauterine viral transmission in a case series of 31 SARS-CoV-2 positive mothers. Abbreviations: nasopharynx (NPH), polymerase chain reaction for SARS-CoV-2 (PCR), immunohistochemistry for SARS-CoV-2 (IHC), not analysed (n/a), positive (pos).
