# Supplementary material for: Intrauterine vertical SARS‐CoV‐2 infection: a case confirming transplacental transmission followed by divergence of the viral genome
Source: BJOG. 2021 Mar 22;128(8):1388–94. doi: 10.1111/1471-0528.16682 (PMC8013698; doi:10.1111/1471-0528.16682)
Supplement: Supplementary file 5 — Table S2. Real time reverse transcriptase quantitative polymerase chain reaction (PCR) analyses with cycle threshold (CT) values for severe acute respiratory syndrome coronavirus‐2 (SARS‐CoV‐2) in maternal, placental and neonatal samples. [file BJO-128-1388-s018.docx]

**Table S2.**

Real time reverse transcriptase quantitative polymerase chain reaction (PCR) analyses with cycle threshold (CT) values for severe acute respiratory syndrome coronavirus-2 (SARS-CoV-2) in maternal samples, placental sample, and neonatal samples.

| **Sample** | **Time of sampling** | **Result** | **CT-value** |
| --- | --- | --- | --- |
| Maternal duo swab (NPH/throat) | Day of delivery | Positive | 26.7 |
| Maternal blood (EDTA-plasma) | Day of delivery | Positive | 31.7 |
| Maternal duo swab (NPH/throat) | Day 9 postpartum | Negative | - |
|  |  |  |  |
| Placenta | Day of delivery | Positive | 13.6 |
|  |  |  |  |
| Neonate NPH swab | DOL 2 | Positive | 27.9 |
| Neonate NPH swab | DOL 5 | Positive | 18.1 |
| Neonate Throat swab | DOL 5 | Positive | 23.5 |
| Neonate NPH swab | DOL 12 | Positive | 36.1 |
| Neonate Throat swab | DOL 12 | Positive | 33.3 |
| Neonate NPH swab | DOL 14 | Positive | 34.5 |
| Neonate Throat swab | DOL 14 | Negative | - |
| Neonate NPH swab | DOL 16 | Positive | 29 |
| Neonate Throat swab | DOL 16 | Negative | - |
| Neonate NPH swab | DOL 20 | Negative | - |
| Neonate Throat swab | DOL 20 | Negative | - |
