# Supplementary material for: Intrauterine vertical SARS‐CoV‐2 infection: a case confirming transplacental transmission followed by divergence of the viral genome
Source: BJOG. 2021 Mar 22;128(8):1388–94. doi: 10.1111/1471-0528.16682 (PMC8013698; doi:10.1111/1471-0528.16682)
Supplement: Supplementary file 6 — Table S3. Variant analysis and annotation of whole‐genome sequencing data from virus isolates obtained from the mother and placenta at delivery and from the neonate at day of life (DOL) 2 and DOL 5. [file BJO-128-1388-s010.docx]

**Table S3.**

Variant analysis and annotation of whole-genome sequencing data from virus isolates obtained from the mother and placenta at delivery and from the neonate at day of life (DOL) 2 and DOL 5. The table shows all identified variants, single-nucleotide polymorphisms (SNP) or multiple-nucleotide polymorphisms (MNP), with corresponding nucleotide position in reference genome of for severe acute respiratory syndrome coronavirus-2 (SARS-CoV-2) (NC_045512), the frequency (%) of the variant in the sequencing data and their gene-based annotation.

| Variant | % variant in the sample | | | | Type of variant | Gene | Type of mutation | Affected AA | Predicted consequence* |
| --- | --- | --- | --- | --- | --- | --- | --- | --- | --- |
|  | Mother | Placenta | DOL 2 | DOL 5 |  |  |  |  |  |
| A107G | 0 | 0 | 80 | 67 | SNP | orf1ab | upstream gene | . | MODIFIER |
| C241T | 100 | 100 | 100 | 100 | SNP | orf1ab | upstream gene | . | MODIFIER |
| C3037T | 100 | 100 | 100 | >99 | SNP | orf1ab | synonymous | Phe924Phe | LOW |
| G9871A | 100 | 100 | >99 | 100 | SNP | orf1ab | synonymous | Thr3202Thr | LOW |
| C14408T | 100 | >99 | 100 | 100 | SNP | orf1ab | missense | Pro4715Leu | MODERATE |
| A23403G | 100 | 100 | 100 | 100 | SNP | S | missense | Asp614Gly | MODERATE |
| A23851G | 100 | 100 | 100 | 100 | SNP | S | synonymous | Leu763Leu | LOW |
| A24292G | 100 | >99 | 100 | 100 | SNP | S | synonymous | Gly910Gly | LOW |
| C26447T | 100 | 100 | 100 | 100 | SNP | E | missense | Ser68Phe | MODERATE |
| G26727T | 100 | 100 | 100 | 100 | SNP | M | missense | Ala69Ser | MODERATE |
| GGG28881AAC | 100 | 100 | 100 | 100 | MNP | N | missense | ArgGly203LysArg | MODERATE |
| C28887T | >99 | >96 | >99 | >99 | SNP | N | missense | Thr205Ile | MODERATE |
| C29666T | 100 | 100 | 100 | 100 | SNP | ORF10 | missense | Leu37Phe | MODERATE |

*Ensemble variation. Calculated variant consequences. http://www.ensembl.org/info/genome/variation/prediction/predicted_data.html
